# Clinical characteristics of congenital heart defects in mild congenital anorectal malformation: single-centre experience

**DOI:** 10.1186/s12887-023-04518-9

**Published:** 2024-01-20

**Authors:** Wei Feng, Minjie Zhang, Jinping Hou, Xiaohong Die, Yi Wang, Rong Liu

**Affiliations:** Department of General & Neonatal Surgery, Children’s Hospital of Chongqing Medical University; National Clinical Research Center for Child Health and Disorders, Ministry of Education Key Laboratory of Child Development and Disorders, Chongqing Key Laboratory of Structural Birth Defect and Reconstruction, Chongqing, China

**Keywords:** Congenital heart defect, Congenital anorectal malformation, Clinical characteristic, Sex difference

## Abstract

**Objective:**

To analyze the clinical characteristics and types of congenital heart defect (CHD) in mild congenital anorectal malformation (CARM), namely the rectoperineal and rectovestibular fistulas.

**Methods:**

The retrospective study of 183 patients with mild CARM was conducted with assessments of demographic information, color Doppler echocardiography results, and follow-up data. We performed an analysis of the clinical characteristics of CHD, grouping them based on sex and type of mild CARM.

**Results:**

Of the 183 patients, rectoperineal fistula occurred in 133 patients (72.7%), while the frequency of CHD was 79.8% (146/183). Ventricular septal defects (VSDs) occur more frequently in patients with rectoperineal fistula compared to those with rectovestibular fistula (1.5% vs. 10%), while the opposite trend was observed for patent ductus arteriosus (PDAs) (39.8% vs. 22.0%). Additionally, males presented higher frequency of PDA (42.7% vs. 26.4%) and self-healing (6 months: 87.2% vs. 42.6%; 12 months: 91.0% vs. 63.2%) than females. However, males had a lower rate of undergoing cardiac surgery (6.4% vs. 17.6%) and a younger median diagnosis age (1 day vs. 9 days).

**Conclusion:**

Our study indicates that there is a necessity for meticulous cardiac assessment and follow-up in neonates diagnosed with mild CARM.

## Introduction

Congenital anorectal malformation (CARM) is a congenital condition involving the anus and rectum, with a prevalence of approximately 3.26 per 10,000 births in the population [1]. Moreover, most CARM patients are known to have other systemic congenital malformations, such as vertebral, spinal cord, cardiac, tracheo-esophageal, and urinary system abnormalities [2, 3]. Although the severity of these malformations is related to the complexity of CARM, congenital heart defects (CHDs) seem to be an exception [4, 5]. Due to the high frequency of associated malformations, thorough screening, including color Doppler echocardiography, is essential for all CARM patients [6–8]. Unfortunately, studies have shown that only 63% of CARM patients receive thorough screening, while incomplete screening observed more frequently in female patients and those with mild CARM (including rectoperineal and rectovestibular fistulas) [2, 9]. Color Doppler echocardiography, in particular, is considered the most valuable of the diagnostic workup. Study reported by Evans-Barns et al. found that patients with mild CARM were significantly less likely to receive thorough screening compared to patients with complex form [2].

CHD is the more common congenital malformation, with an incidence of 10 to 14 cases per 1000 live births in the general population [10–12]. It should be noted that the incidence of CHD differs between males and females, with studies reporting a higher prevalence in females [11–13]. In addition, the incidence of CHD varies depending on the specific type of defect and the sex of the individual. Generally, males are more likely to have complex or severe forms of CHD than females, although the reason for these differences remains unclear [14–16]. The incidence rate of CHD has been reported to range from 9 to 37% in the CARM population [17].

Surgical treatment is required for confirmed CARM, and the associated risks of surgery and anesthesia increase when CHD is present [18]. CHD increases the reintubation rate and operative mortality in patients with CARM [19]. Therefore, routine preoperative screening with color Doppler echocardiography and assessment of cardiac function are crucial [20]. Rectoperineal fistulas are considered a mild form of CARM, and approximately one-third of patients with this malformation also have CHD [5]. However, individuals with mild CARM are often overlooked for CHD screening, and there is a lack of out-of-hospital follow-up studies for patients diagnosed with mild CARM combined with CHD.

Although much attention has been given to complex forms of CARM, there is currently limited research concerning mild CARM patients with CHD. The frequency and clinical outcomes, as well as potential sex-related differences, in mild CARM patients with CHD, remain unknown. Additionally, the clinical characteristics of CHD in different types of mild CARM are also unclear. Therefore, this study aimed to investigate the clinical characteristics of different types of mild CARM patients with CHD, including sex-associated differences.

## Methods

This study was approved by the Institutional Research Ethics Board of Children’s Hospital affiliated Chongqing Medical University (Date: 2021/No: 391).

We retrospectively collected data of 183 patients with mild CARM during the period from January 2015 to December 2019 in General & Neonatal Surgery Department of Children’s Hospital affiliated Chongqing Medical University. The following variables were collected: type of mild CARM, sex, diagnosis age, echocardiographic results at the ages of diagnosis, 6 months, and 12 months (follow-up echocardiography was performed in CHD patients using PHILIPS EPIQ7C ultrasonic diagnostic instrument [China]). Only patients who had complete clinical data and cooperated with the follow-up process were included. Additionally, all patients received Color Doppler echocardiography conducted by specialized technicians. For patients who had abnormal echocardiographic results, we consulted with our team of cardiologists to evaluate the need for further diagnostic techniques, interventional cardiac catheter or surgical management. Being the largest tertiary pediatric referral center in the southwest region of China, our hospital follows standardized diagnosis and treatment processes for CHD.

### Definitions

According to the Krickenbeck classification [21], patients with rectoperineal fistula or rectovestibular fistula were classified as mild CARM; other types of CARM were classified as complex form [20].

Referring to the echocardiography results, we also distinguished three forms of CHD based on the presence and severity: no CHD (no abnormality), minor CHD (incomplete foramen ovale closure with a significant shunt, secundum atrial septal defect, and/or small ventricular septal defect), and major CHD (the remaining defects) [20, 22, 23]. Furthermore, we defined self-healing of CHD as: the abnormal cardiac anatomy, which met the diagnostic criteria for CHD, disappeared spontaneously as confirmed by color Doppler echocardiography during the follow-up period.

Furthermore, we assessed the presence of multiple congenital disorders (MCD). MCD was defined as the presence of three or more congenital malformations, including CARM, or if they had a syndrome that was confirmed by a clinical geneticist [1, 2, 20].

### Statistical analyses

Data were analyzed with SPSS 22.0 (IBM SPSS for Windows, IBM Corporation, Somers, NY, the USA) and GraphPad Prism 8.0 (GraphPad Software Inc., San Diego, CA, USA). Categorical data were expressed by number and percentage (n and %), and analyzed by Fisher’s exact test or chi-squared test, as appropriate. In addition, numerical data were expressed as quartile spacing with Mann-Whitney test. The level of statistical significance was set at a probability of < 0.05.

## Results

### General data

Of the 183 cases (male: 87, female: 96), 133 cases (72.7%) were identified as rectoperineal fistula, while the remaining 50 cases (27.3%) were diagnosed as rectovestibular fistula. Notably, rectovestibular fistula was observed exclusively in females, while the male-to-female ratio for rectoperineal fistula was 2.6 (96:37). Among the cases, 146 (79.8%) were found to have CHD, with 17 cases (9.3%) receiving cardiac surgery and 82 cases (44.8%) classified as major CHD. The diagnosis age of CHD ranged from 1 to 67 days, with 119 cases (65.0%) were diagnosed within the first week and 165 cases (90.2%) were diagnosed within the first month (Table 1). Additionally, 52 cases (28.4%) were identified as having MCD. Besides heart defects, the other associated congenital malformations were genitourinary (28 cases), spinal/vertebral (19 cases), musculoskeletal (3 cases), gastrointestinal (3 case), chromosomal (1 cases) and brain (1 cases) anomalies. During follow-up period, no patients died from CHD.

**Table 1:** Patient characteristics

Clinical data	Number of patients (%)
Total patients	183
Type of mild CARM	
Rectoperineal fistula	133(72.7)
Rectovestibular fistula	50(27.3)
Sex	
Female	96(52.5)
Male	87(47.5)
Age at CHD diagnosis (days): 0 ~ 67	
Within 1 week	119 (65.0)
Within 1 month	165 (90.2)
More than 1 month	18 (9.8)
Forms of CHD#	
No	37(20.2)
Minor	64(35.0)
Major	82(44.8)
Cardiac surgery	17(9.3)
MCD	
No	131(71.6)
Yes	52(28.4)

CARM, congenital anorectal malformation; CHD, congenital heart defect; MCD, multiple congenital disorders. Based on the severity of CHD, minor CHD includes the following defects: incomplete foramen ovale closure with a significant shunt, secundum atrial septal defect, and/or small ventricular septal defect; and other defects of CHD were classified as major form

### Frequency of specific defect types in CHD

**Fig. 1:**
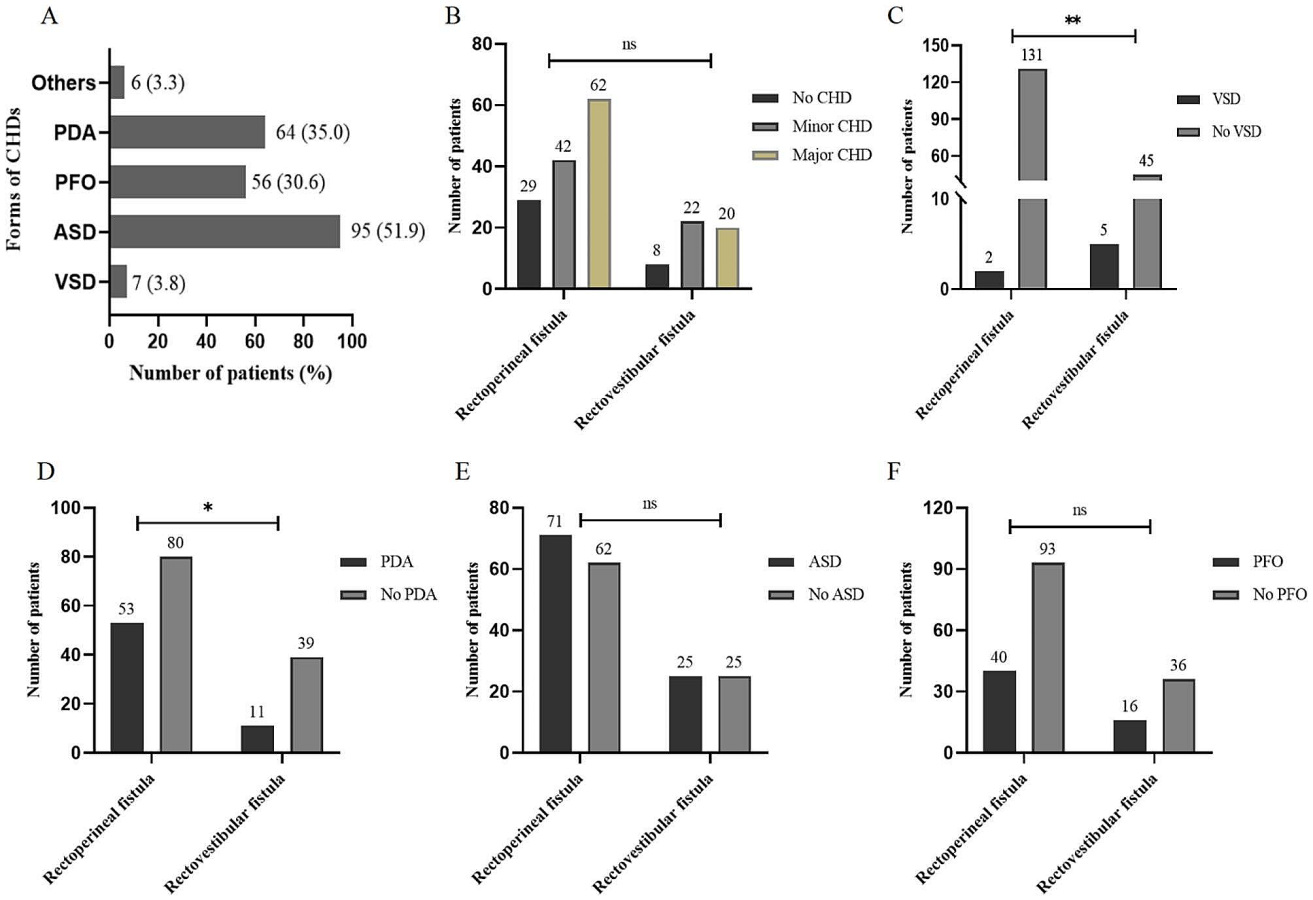
Distribution of specific defects of CHD in different types mild CARM. Note: ns, *P* > 0.05; *, *P* < 0.05; **, *P* < 0.01

Among these cases diagnosed with CHD, the frequency of specific defects was as follows (Fig. 1A): ventricular septal defect (VSD) was present in 7 cases (3.8%, 5 in perimembranous type and 2 in muscular type, diameter [mean ± standard deviation]: 5.30 ± 2.88 mm), atrial septal defect (ASD) in 95 cases (51.9%), patent foramen ovale (PFO) in 56 cases (30.6%), patent ductus arteriosus (PDA) in 64 cases (35.0%), and other types of CHD (1 in congenital double aortic arch, 1 in dextrocardia, 1 in aortic constriction, 1 in tetralogy of Fallot, and 2 in aortic arch dysplasia) in 6 cases (3.3%). Of these patients, 3 cases received anti-failure measures for hemodynamic instability/heart failure and 1 case with VSD developed infective endocarditis, as shown in Table 2.

**Table 2:** Types of CHD versus diagnosis age and clinical outcomes

Type of CHD	Diameter (mm)*	Diagnosis age (day)*	Self-healing within 12 months (cases)	Cardiac surgery within 12 months (case)^#^	Hemodynamically unstable/heart failure need anti-failure measures (case)
VSD (*n* = 7)	5.30 ± 2.88	27 ± 10.2	4	2	1
Perimembranous type (*n* = 5)	5.00 ± 3.11	7 (2, 50.5)	2	2	1
Muscular type (*n* = 2)^ǂ^	6.8/4.6	21/61	2	0	0
ASD (*n* = 95)	4.61 ± 2.07	1 (0, 4.8)	73	11	1
PFO (*n* = 56)	-	2 (1, 17.5)	50	2	0
PDA (*n* = 64)	-	1 (0, 1)	54	5	0
Others (*n* = 6)	-	1.5 (1, 2)	0	3	1

^*^ Numerical data were assessed for normality by the Shapiro-Wilk test: if matched, they were expressed as mean ± standard deviation; if not, they were expressed as median (quartile spacing)

^#^ Note: 2 cases underwent surgery for ASD + PDA, 2 cases for VSD + PFO, 1 case for aortic constriction + PDA, 1 case for congenital double aortic arch + VSD.

^ǂ^ the diameters of the 2 cases were 6.8 and 4.6 mm, respectively; and the diagnosis age was 21 and 61 days, respectively

By analyzing the frequency of CHD in the two types of mild CARM, the following findings were obtained. The proportions of no CHD, minor CHD, and major CHD were 21.8% (29), 31.6% (42), and 46.6% (62) in patients with rectoperineal fistula, respectively; while the proportions of no CHD, minor CHD, and major CHD were 16% (8), 44% (22), and 40% (20) in patients with rectovestibular fistula, respectively; no statistical significance was observed between the two groups (P>0.05, Fig. 1B). Additionally, the frequency of VSD (1.5% vs. 10%, Fig. 1C) was significantly lower in patients with rectoperineal fistula compared to those with rectovestibular fistula, whereas the opposite was true for PDA (39.8% vs. 22.0%, Fig. 1D) (both *P* < 0.05). However, significant differences were not observed in the frequency of ASD (53.4% vs. 50.0%, Fig. 1E) or PFO (30.1% vs. 32.0%, Fig. 1F) between the two groups (both P>0.05).

**Fig. 2:**
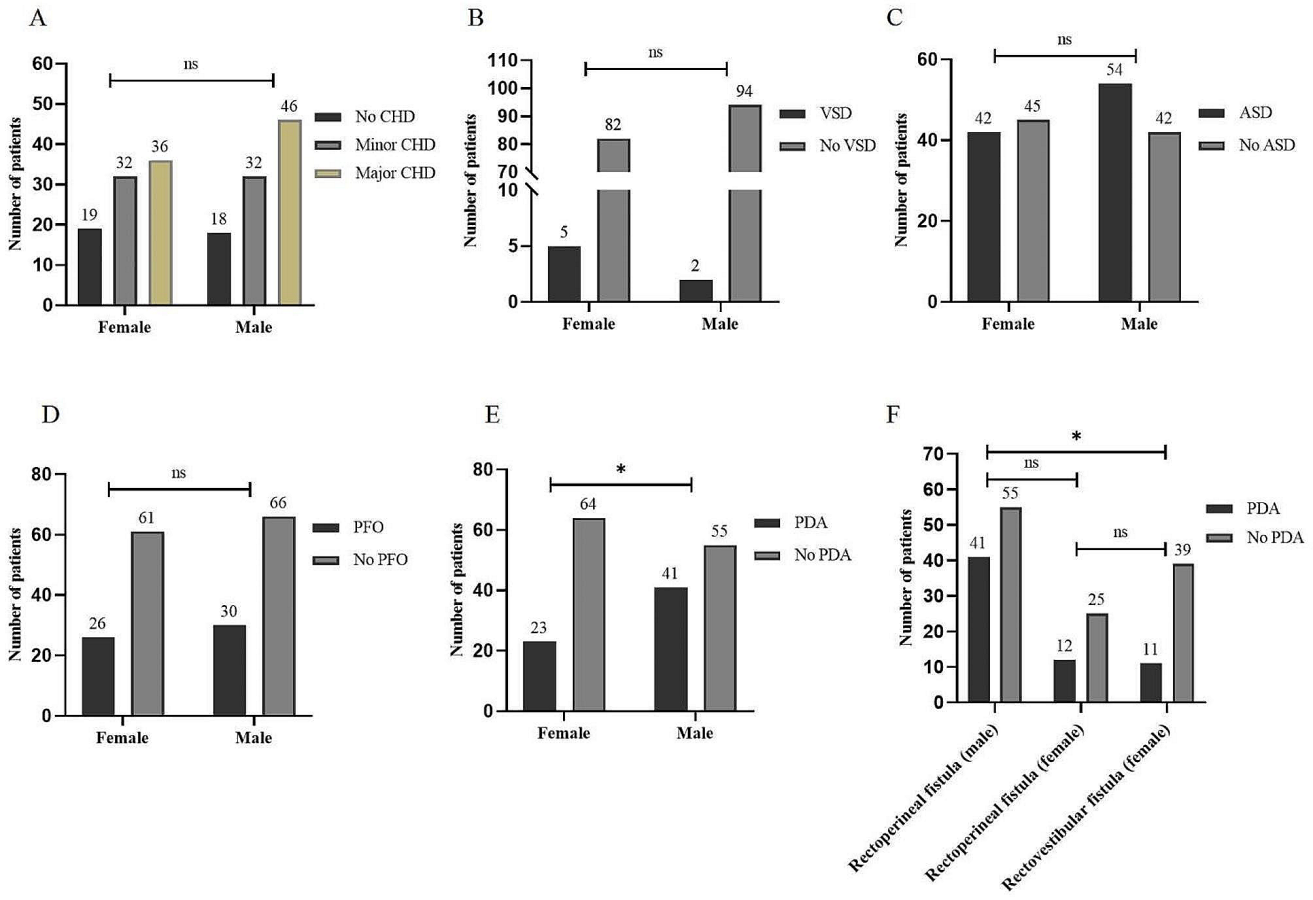
Sex differences in the frequency of specific types of CHD. Note: ns, *P* > 0.05; *, *P* < 0.05

CHD was diagnosed in 81.25% (78) of males and 78.16% (68) of females. The proportions of no CHD, minor CHD, and major CHD in males were 21.8% (19), 41.4% (36), and 36.8% (32), respectively; while the proportions of no CHD, minor CHD, and major CHD in females were 18.8% (18), 47.9% (46), and 33.3% (32), respectively; no sex difference observed in the severity of CHD (P>0.05, Fig. 2A). Furthermore, there was no sex differences in the frequency of VSD, ASD, and PFO (P>0.05, Fig. 2B-E). However, males were at greater risk of developing PDA compared to females (42.7% vs. 26.4%, *P* < 0.05). To clarify the cause of variation in the frequency of PAD, we conducted a subgroup analysis and found that the significant difference of PDA frequency was existed only between males with rectoperineal fistula and females with rectovestibular fistula (Fig. 2F).

### Diagnosis age of CHD

**Fig. 3:**
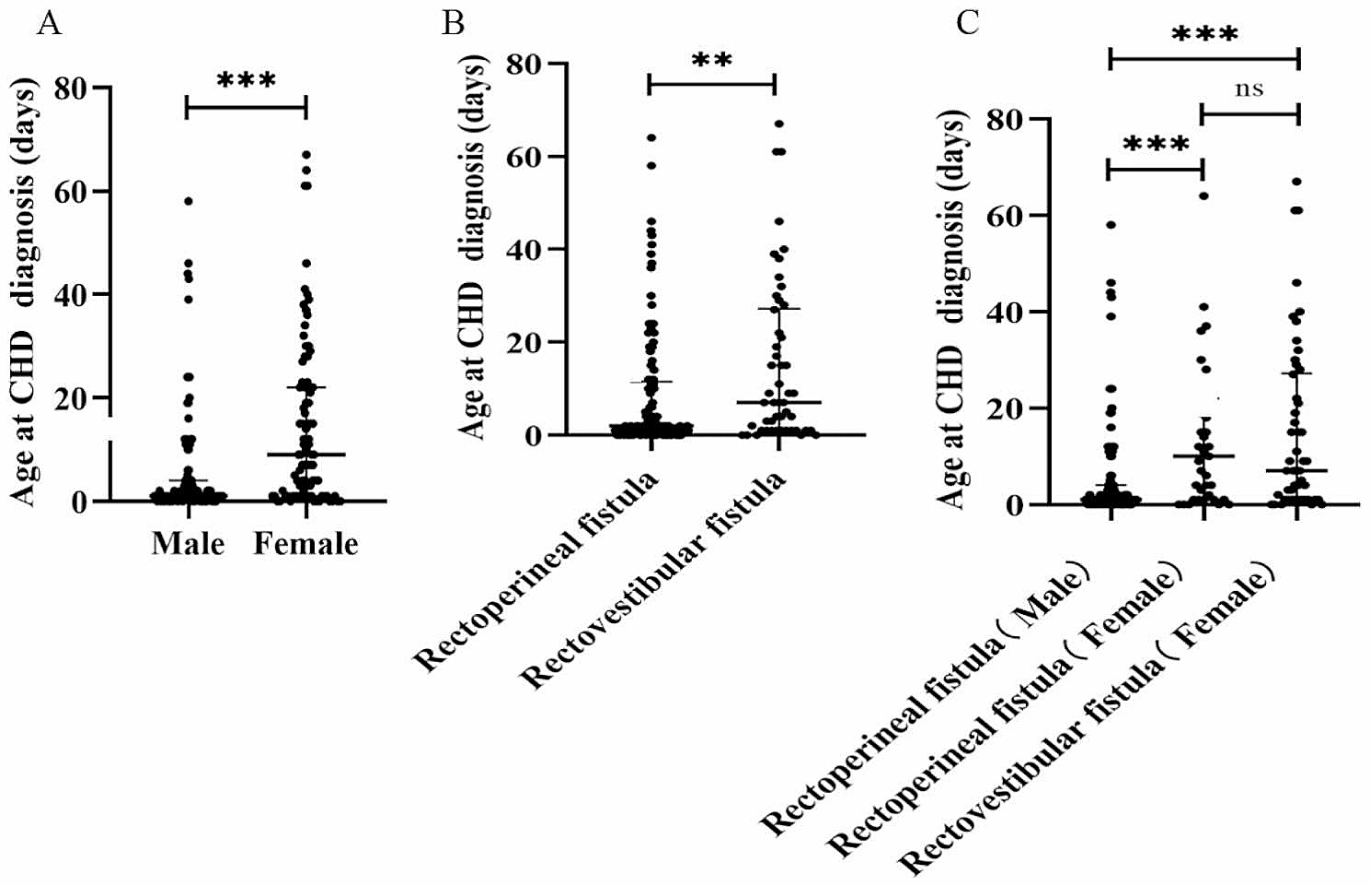
Differences in diagnosis age of CHD based on sex and type of mild CARM. Note: ns, *P* > 0.05; **, *P* < 0.01; ***, *P* < 0.001

The diagnosis age of CHD was significantly lower in males than that in females (median: 1 day vs. 9 days, *P* < 0.05, Fig. 3A). Additionally, the diagnosis age of CHD in patients with rectoperineal fistula was significantly lower than in those with rectovestibular fistula (median: 2 days vs. 7 days, *P* < 0.05, Fig. 3B). It is important to note that rectovestibular fistula, a condition exclusive to females, may have introduced a confounding effect on the results. Our subgroup analysis of rectoperineal fistula found that the diagnosis age of CHD in males was significantly lower than that in females, including rectovestibular and rectoperineal fistulas (median: 1 day vs. 7 days and 10 days, both *P* < 0.05, Fig. 3C). However, in females, there was no significant difference in the diagnosis age of CHD between different types of mild CARM (*P* > 0.05, Fig. 3C). Furthermore, the diagnosis age for specific defect types of CHD is shown in Table 2, it is interesting to note that diagnosis age of VSD was significantly late.

### Self-healing and surgery of CHD

Mild CARM patients with CHD who do not cause significant symptoms, such as significantly decreased activity tolerance, dysontogenesis, cyanosis, and related pneumonia, do not require cardiac surgery and typically exhibit self-healing. Of 146 patients with CHD, 1 patient received cardiac surgery within 6 months, while 48 patients (32.9%) still had CHD at 6 months, with a self-healing rate of 66.4%. At 12 months, we found that 17 patients received cardiac surgery, and 15 patients (10.3%) still had CHD, with a self-healing rate of 78.1%. The self-healing rates of CHD at 6 months (males: 87.2%; females: 42.6%) and 12 months (males: 91.0%; females: 63.2%) were significantly associated with sex (*P* < 0.05, Table 3). The self-healing rate of minor CHD was 70.3% at 6 months and 85.9% at 12 months, while that of major CHD was 63.6% at 6 months and 72.0% at 12 months; there was no correlation between self-healing rates and severity of CHD (Table 3). Additionally, patients with rectoperineal fistula had significantly higher self-healing rate compared to those with rectovestibular fistula, both at 6 months (81.7% vs. 28.6%) and 12 months (88.5% vs. 52.4%). To further investigate the potential influence of sex on the disparities in self-healing rate of CHD within different types of mild CARM, a more comprehensive comparison was conducted. It revealed that the self-healing rate of CHD was notably higher in males compared to females, both rectoperineal and rectovestibular fistulas (Table 3).

**Table 3:** Differences in self-healing rates of CHD based on sex, form of CHD and type of mild CARM

Variable*	Self-healing at 6 months#		*P* value	Self-healing at 12 months#		*P* value
	Yes (*n* = 97)	No (*n* = 49)		Yes (*n* = 114)	No (*n* = 32)	
Sex			< 0.001			< 0.001
Female	29 (42.6)	39 (57.4)		43 (63.2)	25 (36.8)	
Male	68 (87.2)	10 (12.8)		71 (91.0)	7 (9.0)	
Form of CHD			0.558			0.121
Minor	45 (70.3)	19 (29.7)		55 (85.9)	9 (14.1)	
Major	52 (63.4)	30 (36.6)		59 (72.0)	23 (28.0)	
Type of CARM			< 0.001			< 0.001
Rectoperineal fistula	85 (81.7)	19 (18.3)		92 (88.5)	12 (11.5)	
Rectovestibular fistula	12 (28.6)	30 (71.4)		22 (52.4)	20 (47.6)	
Type of CARM based on sex			< 0.001			< 0.001
Rectoperineal fistul (male)	68 (87.28)	10 (12.8)		71 (91.0)	7 (9.0)	
Rectoperineal fistula (female)ǂ	17 (65.4)	9 (34.6)		21 (80.8)	5 (19.2)	
Rectovestibular fistula (female)ǂ	12 (28.6)	30 (71.4)		22 (52.4)	20 (47.6)	

* Categorical data were expressed by n (%), and the chi-squared test was used for comparison

ǂ Compared with group of rectoperineal fistul (male), the distribution of frequencies was statistically significant

# Note: Patients who underwent cardiac surgery were considered non-self-healing; 1 case received surgery within 6 months, and 17 cases received surgery within 12 months

**Fig. 4:**
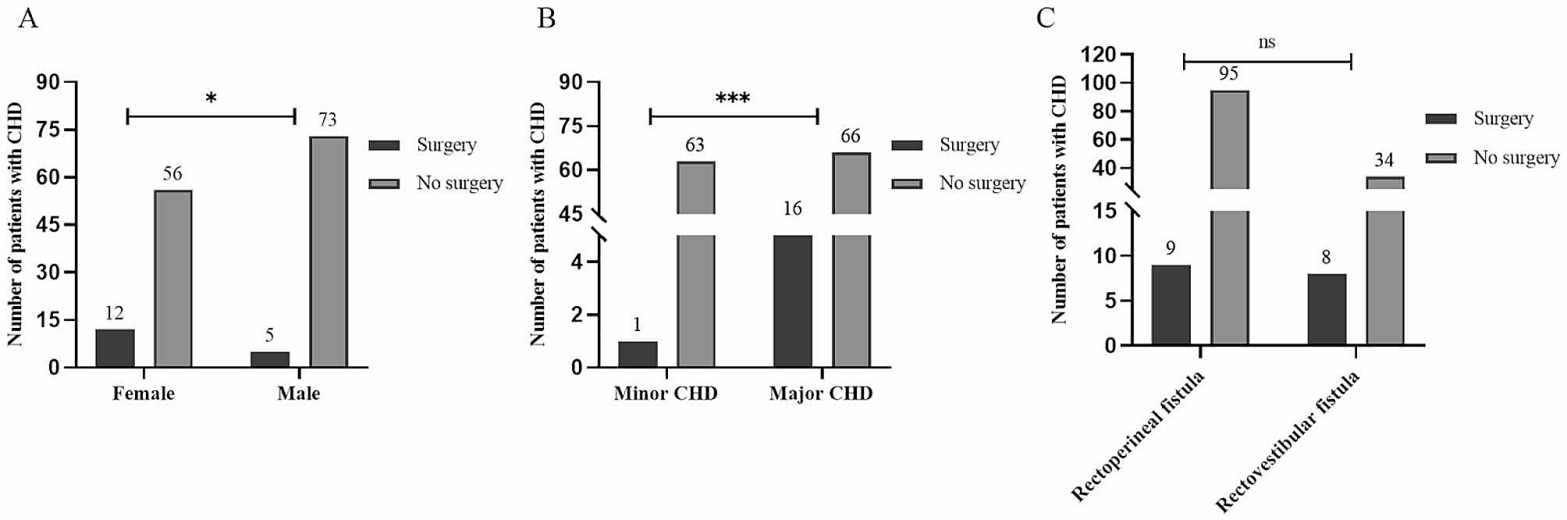
Differences in surgical rate base on sex, as well as severity of CHD and type of mild CARM. Note: ns, *P* > 0.05; *, *P* < 0.05; ***, *P* < 0.001

Among the follow-up cases, 17 (5 males and 12 females) patients underwent invasive cardiac procedures: catheter intervention in 8, open heart surgery in 8, and thoracoscopic closure in 1. All patients who underwent cardiac surgery recovered well except one patient who developed occluder thrombosis after catheter intervention for ASD. Notably, the invasive intervention rate among patients with CHD was significantly lower in males compared to females (6.4% vs. 17.6%, *P* < 0.05, Fig. 4A). The surgical rate among patients with major CHD was found to be 19.5%, a statistically significant increase compared to patients with minor CHD (19.5% vs. 1.6%, *P* < 0.05, Fig. 4B). However, there was no significant difference in cardiac surgery rates between the two types of mild CARM (8.7% vs. 19.0%, *P* > 0.05, Fig. 4C). From the above analysis of self-healing and surgery, it can be seen that the clinical outcomes of CHD differ between the sexes.

### Comparison of MCD occurrence

**Fig. 5:**
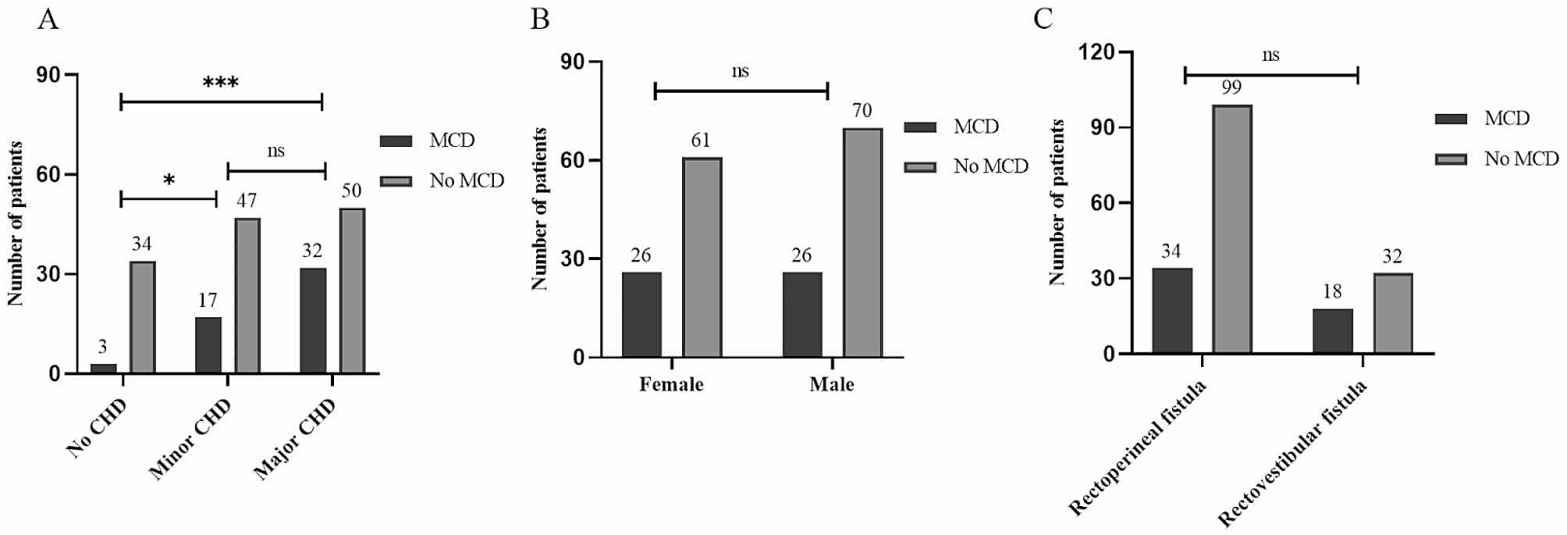
Differences in the frequency of MCD based on sex, as well as severity of CHD and type of mild CARM. Note: ns, *P* > 0.05; ***, *P* < 0.001

We analyzed to determine if patients with major CHD exhibit a greater likelihood of developing MCD. It revealed that the frequency of MCD among patients with major CHD was 39.0%, which was higher compared to minor CHD (26.6%), albeit without statistical significance (*P*>0.05). However, the frequency of MCD in patients without CHD was significantly lower than that in patients with CHD (including minor and major CHDs) (8.1% vs. 26.6% and 39.0%, both *P* < 0.05, Fig. 5A). There was no statistically significant difference in the frequency of MCD based on sex (female: 29.9%; male: 27.1%) or the type of mild CARM (rectovaginal fistula: 25.6%; rectovestibular fistula: 36.0%) (both *P* > 0.05, Fig. 5B-C).

## Discussion

CARM is a rare congenital malformation of the gastrointestinal tract characterized by the complete or partial absence of the anorectal canal at birth, with or without a fistula. It is often associated with other system malformations, of which the cardiovascular system is more common. As a rule, a complex form of CARM is evident, and pediatric clinicians preferred to perform thorough screening for these patients [2–4]. Unfortunately, in case of a mild form of CARM, the screening mostly depends on the clinician preferences, which may be easily overlooked. Thorough screening can identify associated malformations early, help to ensure the safety of surgery, and formulate the optimal treatment plan and postoperative individualized management. Screening for CHD, in particular, is considered the most variable aspect of the diagnostic workup.

The frequency of CHD in CARM cases varies across different research studies. Study from India showed that the frequency of CHD in CARM patients was 50%, and the most common presentation was ASD, followed by VSD and PDA [24]. But another study reported that the frequency of CHD in CARM patients was 22%, and the most frequent defect was VSD [25]. Regarding the frequency of CHD in mild CARM patients, Jonker JE et al. reported that it was 16%, of which 9% were diagnosed as minor CHD and 7% were diagnosed as major CHD [20]. In our study, the frequency of CHD in mild CARM patients was 79.8%, which is significantly higher than that reported in other studies. This may be due to the fact that we always insist on performing echocardiography screening for every patient with CARM to avoid as many missed diagnoses as possible, whether it’s a complex or a mild CARM. However, none of these studies conducted a distinction between different types of mild CARM. In our study, we observed that the highest frequency of specific CHD was ASD, both in patients with rectovestibular and rectoperineal fistulas, accounting for 53.4% and 50.0%, respectively. Patients with rectoperineal fistula had a higher probability of developing PDA, while patients with rectovestibular fistula were more prone to VSD. Several studies have demonstrated a significantly higher frequency of CHD in CARM patients compared to the general population [17, 22, 26]. The presence and severity of CHD affect the choice of surgical methods and timing not only for mild CARM, but also for complex form [27, 28]. For rectoperineal fistula, we chose the following surgical method based on the presence and severity of CHD: (1) no or minor CHD: cutback anoplasty with or without mobilized the rectum end inside the center of sphincter; (2) major CHD: routine dilations of the fistula to permit egress of stool (typically Hegar size > 5 mm) and then delayed repair with cutback anoplasty. For rectovestibular fistula, the following principles are our preference: (1) no or minor CHD: modified posterior sagittal anorectoplasty (saving the perineal body); (2) major CHD: routine dilations of the fistula to permit egress of stool and then delayed repair with modified posterior sagittal anorectoplasty, or three-staged repair (transverse-loop colostomy, modified posterior sagittal anorectoplasty, and colostomy) for those abdominal distension not relieved after anal dilatation. Consequently, we recommend the routine utilization of color Doppler echocardiography in mild CARM patients to assess the presence and severity of CHD and to ensure the safety of surgical procedure and postoperative care.

Previous population-based study has documented the presence of sexual dimorphism in various forms of CHD, with a higher prevalence of ASD and ASD in females, while a male predominance in aortic stenosis, coarctation of aorta, transposition of the great arteries, TOF, and double outlet right ventricle [29]. Our study found that males had a higher likelihood of having PDA, while no significant differences were observed among the other forms of CHD. Rectovestibular fistula is the most important CARM in females, presenting with the anal opening is located in the vestibule of the female genitalia, far away from the anal sphincter [7]. Considering that all cases of rectovestibular fistula were observed exclusively in females, our subgroup analysis of rectoperineal fistula revealed that no sex differences existed between the types of CHD. These findings suggest that there is no sex disparity in the types of mild CARM associated with CHD, a finding that has not been previously reported in other studies.

There are few studies on the diagnosis age of CHD in mild CARM patients, and our study showed that the diagnosis age of CHD ranged from 0 to 67 days, with 119 cases (65%) diagnosed within 1 week and 165 cases (90.2%) diagnosed within 1 month. Furthermore, the diagnosis age of CHD tends to be higher in females compared to males, and this trend was also observed in the subgroup analysis of rectoperineal fistula. However, there was no statistical difference in the diagnosis age of CHD between rectovestibular fistula and rectoperineal fistula in females. It means that there is a correlation between diagnosis age and sex, but not the specific type of mild CARM. This phenomenon could potentially be attributed to the timing of mild CARM diagnosis, as patients typically do not undergo routine echocardiography at birth and are usually assessed for CHD when mild CARM is detected or when they are hospitalized for symptoms associated with mild CARM. Complex CARM encompasses various types of lesions, and the absence of an anal opening in the anal crypt is easily identifiable at birth. However, most mild CARM patients often do not exhibit prominent symptoms apart from chronic constipation, and inexperienced physicians may overlook mild CARM, resulting in delayed diagnosis [30–32]. This study did not compare CHD among those with complex CARM as it was beyond the scope of this study. We are not aware if males exhibit more pronounced and earlier clinical manifestations compared to females [33]. However, it is important to note that this is merely speculative and requires further investigation through subsequent studies.

The self-healing rate of CHD was 66.4% at 6 months and 78.1% at 12 months in our study. Notably, these rates were found to be significantly higher in males compared to females. Furthermore, females had significantly higher rate of undergoing cardiac surgery compared to males. These results are consistent with the European Heart Survey database of adult CHD, which suggests that females may have a higher prevalence of limited cardiac function compared to males, possibly related to hormone level [34, 35]. Based on the observation that females tend to receive a diagnosis of CHD at a later age compared to males, and exhibit lower rates of self-healing at 6 and 12 months, we propose the implementation of enhanced cardiac evaluation and closer monitoring for females with mild CARM. It is widely accepted that minor CHD is more likely to heal spontaneously compared to major CHD, and this is supported by our study, although not statistically significant, which may be due to inadequate sample size. Analysis of cardiac surgery rates in patients with different severity of CHD showed that the surgery rate in patients with major CHD was significantly higher than that in patients with minor CHD, aligning with our anticipated results. Additional, the self-healing rates of CHD at 6 months and 12 months in patients with rectovestibular fistulas were significantly lower than that in patients with rectovaginal fistula, but there was no correlation between cardiac surgery rates. It also indicates that there is a necessity for exceptional cardiac assessment and follow-up in females with mild CARM.

It’s known that severe CHD often coexists with MCD. Study indicates that up to 70% of patients with CARM also exhibit malformations in other bodily systems [36]. Recently, it has been reported that CARM patients with extracardiac malformations have a notable frequency of combined severe CHD, with each additional extracardiac malformation increasing the probability of severe CHD by 3.85 times [37]. Similar results were obtained in this study, that the frequency of MCD was not associated with a specific type of mild CARM or sex, but with the presence of CHD. Specifically, the prevalence of MCD was observed to be 39% and 26.6% in patients with major and minor CHDs, respectively. Although statistical significance was not achieved, a discernible trend is apparent, potentially attributable to the limited size of our sample.

To further provide early and thorough screening for suspicious or confirmed neonates, we have established a circular linkage mechanism with hospitals at all levels to enable timely referral to our hospital if malformations are detected. Currently, our hospital actively participates in the construction of regional medical consortia, and carries out prenatal screening of CARM utilizing telemedicine cloud platform in collaboration with obstetrics department. Then, for suspicious cases, pediatricians provide perinatal professional consultation to the family numbers with the help of “Internet +” technology, and carried out seamless referral of confirmed cases after delivery [38]. Overall, by collecting comprehensive clinical data, conducting specialized echocardiography, and following standardized treatment processes, it helps to to provide the best possible care and outcomes for these patients.

### Limitation

We should acknowledge that this analysis was a single-center retrospective study with a relatively small cohort and did not necessarily represent the entire population of mild CARM, further research with a larger prospective cohort is necessary to validate the results. Furthermore, our hospital is situated in the economically disadvantaged western region of China. Consequently, it is plausible that a considerable number of patients with mild CARM may not have sought medical attention due to their parents’ limited understanding of the ailment. Finally, it is important to highlight that this study exclusively focuses on patients with mild CARM, and the specific clinical attributes of complex CARM associated with CHD remain inadequately elucidated. Our research team intends to undertake additional investigations in the subsequent phase.

## Conclusion

This is the largest study in China to analyze the clinical characteristics and sex disparities of CHD among patients with mild CARM. Our study found that ASD is the most prevalent anomaly (51.9%), followed by PDA, PFO, and VSD. The frequency of VSD was significantly lower in patients with rectoperineal fistula compared to those with rectovestibular fistula, while the opposite trend was observed for PDA. Furthermore, patients with rectoperineal fistula were diagnosed with CHD at an earlier age compared to those with rectovestibular fistula. Regarding sex differences, the males had higher rates of PDA occurrence and self-healing of CHD compared to females, but lower cardiac surgery rate and younger diagnosis age. In short, our investigation has identified clinical attributes of CHD with the specific type of mild CARM and sex, further research is warranted to validate these differences and their application in clinical practice. Our study indicates that meticulous cardiac examination, diagnostic work up and follow-up is a necessity among in neonates diagnosed with mild CARM.

## Data Availability

The datasets generated and analyzed during the current study are not publicly available due to the ongoing analysis in other directions but are available from the corresponding author (Yi Wang and Rong Liu) on reasonable request.
